# Supplementary material for: Determining composition of micron-scale protein deposits in neurodegenerative disease by spatially targeted optical microproteomics
Source: eLife. 2015 Sep 29;4:e09579. doi: 10.7554/eLife.09579 (PMC4630677; doi:10.7554/eLife.09579)
Supplement: Supplementary file 1. — DOI: http://dx.doi.org/10.7554/eLife.09579.013 [file elife09579s001.pdf]

Table ST1: All proteins identified by STOMP from TgCRND8 mouse plaques

Bait	Prey	PreyGene	IP	Spec	Spec Sum	Num Rep	Prob	iProb	Ctrl Counts	AvgP	MaxP	SAINT score
amyloid	P60879	SNP25_MOUSE_1	STOMP1|STOMP2	5|36	41	2	0.56	0.11|1.00	3|0.5545	1		1.00
amyloid	Q8BH59	CMC1_MOUSE_1	STOMP1|STOMP2	30|25	55	2	1	1.00|1.00	2|4	1	1	1.00
amyloid	P63017	HSP7C_MOUSE_1	STOMP2	21	21	1	1	1	2|0.9990	0.999		1.00
amyloid	O08599	STXB1_MOUSE_1	STOMP1|STOMP2	37|42	79	2	1	1.00|1.00	11|20	0.9965	0.998	1.00
amyloid	Q9CZU6	CISY_MOUSE_1	STOMP1|STOMP2	13|23	36	2	0.99	0.99|1.00	3|0.9905	0.996		1.00
amyloid	P39053	DYN1_MOUSE_1	STOMP1|STOMP2	5|45	50	2	0.49	0.00|1.00	7|0.4975	0.995		1.00
amyloid	Q62261	SPTB2_MOUSE_1	STOMP1|STOMP2	34|57	91	2	0.99	0.99|1.00	11|15	0.995	1	1.00
amyloid	P50516	VATA_MOUSE_1	STOMP1|STOMP2	13|38	51	2	0.75	0.50|0.99	7|0.7440	0.993		0.99
amyloid	Q8K2B3	DHSA_MOUSE_1	STOMP1|STOMP2	15|14	29	2	0.99	0.99|0.99	2|0.9905	0.993		0.99
amyloid	P46660	AINX_MOUSE_1	STOMP1|STOMP2	2|32	34	2	0.51	0.00|0.98	6|0.4930	0.984		0.98
amyloid	Q9D0M3	CY1_MOUSE_1	STOMP1|STOMP2	21|17	38	2	0.99	0.98|0.98	6|6	0.983	0.984	0.98
amyloid	P17710	HXK1_MOUSE_1	STOMP1|STOMP2	14|26	40	2	0.98	0.97|1.00	4|3	0.9815	0.997	1.00
amyloid	P11499	HS90B_MOUSE_1	STOMP1|STOMP2	7|17	24	2	0.85	0.70|0.98	2|0.8425	0.981		0.98
amyloid	P52480	KPYM_MOUSE_1	STOMP1|STOMP2	15|41	56	2	0.7	0.45|0.98	9|0.7155	0.981		0.98
amyloid	Q9Z1G4	VPP1_MOUSE_1	STOMP1|STOMP2	13|23	36	2	0.95	0.90|0.98	5|0.9395	0.981		0.98
amyloid	Q8VDN2	AT1A1_MOUSE_1	STOMP1|STOMP2	35|52	87	2	0.98	0.97|0.99	13|20	0.98	0.994	0.99
amyloid	Q9R0K7	AT2B2_MOUSE_1	STOMP2	14	14	1	0.97	0.97	2|0.9720	0.972		0.97
amyloid	P00405	COX2_MOUSE_1	STOMP1|STOMP2	8|13	21	2	0.97	0.95|0.97	2|0.9625	0.971		0.97
amyloid	P63101	1433Z_MOUSE_1	STOMP2	18	18	1	0.97	0.97	5|0.9680	0.968		0.97
amyloid	P80314	TCPB_MOUSE_1	STOMP2	13	13	1	0.95	0.95	3|0.9540	0.954		0.95
amyloid	Q6PIE5	AT1A2_MOUSE_1	STOMP1|STOMP2	14|18	32	2	0.92	0.91|0.95	6|0.9320	0.953		0.95
amyloid	Q9DCT2	NDUS3_MOUSE_1	STOMP2	10	10	1	0.95	0.95	2|0.9480	0.948		0.95
amyloid	Q91VD9	NDUS1_MOUSE_1	STOMP1|STOMP2	4|20	24	2	0.5	0.03|0.95	5|0.4895	0.946		0.95
amyloid	P05213	TBA1B_MOUSE_1	STOMP1|STOMP2	15|24	39	2	0.95	0.92|0.97	4|9	0.945	0.972	0.97
amyloid	P63038	CH60_MOUSE_1	STOMP2	21	21	1	0.95	0.95	8|0.9450	0.945		0.95
amyloid	P50396	GDIA_MOUSE_1	STOMP1|STOMP2	5|15	20	2	0.73	0.52|0.94	2|0.7295	0.943		0.94
amyloid	Q9DC69	NDUA9_MOUSE_1	STOMP1|STOMP2	8|15	23	2	0.94	0.90|0.99	2|3	0.943	0.989	0.99
amyloid	P07901	HS90A_MOUSE_1	STOMP1|STOMP2	7|23	30	2	0.51	0.12|0.94	7|0.5300	0.938		0.94
amyloid	P09411	PGK1_MOUSE_1	STOMP2	14	14	1	0.93	0.93	5|0.9340	0.934		0.93
amyloid	Q6PIC6	AT1A3_MOUSE_1	STOMP1|STOMP2	27|37	64	2	0.93	0.87|0.98	16|13	0.9275	0.984	0.98
amyloid	P56564	EAA1_MOUSE_1	STOMP1|STOMP2	7|10	17	2	0.88	0.84|0.92	2|0.8800	0.92		0.92
amyloid	P62204	CALM_MOUSE_1	STOMP2	16	16	1	0.92	0.92	0.918	0.918		0.92

Bait	Prey	PreyGene	IP	Spec	Spec Sum	Num Rep	Prob	iProb	Ctrl Counts	AvgP	MaxP	SAINT score
amyloid	Q9CZ13	QCR1_MOUSE_1	STOMP1|STOMP2	19|27	46	2	0.85	0.81|0.91	11|0.8580	0.908		0.91
amyloid	P84078	ARF1_MOUSE_1	STOMP2	17	17	1	0.91	0.91	0.907	0.907		0.91
amyloid	Q64332	SYN2_MOUSE_1	STOMP1|STOMP2	7|23	30	2	0.54	0.16|0.91	7|0.5310	0.905		0.91
amyloid	P43006	EAA2_MOUSE_1	STOMP1|STOMP2	52|27	79	2	0.89	1.00|0.81	25|3	0.9045	0.998	1.00
amyloid	P68369	TBA1A_MOUSE_1	STOMP1|STOMP2	115|224	339	2	0.9	0.86|0.95	33|98	0.904	0.953	0.95
amyloid	P58252	EF2_MOUSE_1	STOMP1	12	12	1	0.9	0.9	4|0.9020	0.902		0.90
amyloid	Q04447	KCRB_MOUSE_1	STOMP1|STOMP2	27|57	84	2	0.9	0.82|0.98	8|22	0.9015	0.979	0.98
amyloid	Q05920	PYC_MOUSE_1	STOMP1|STOMP2	17|5	22	2	0.5	0.90|0.12	5|0.5100	0.901		0.90
amyloid	Q99LC3	NDUAA_MOUSE_1	STOMP1|STOMP2	8|10	18	2	0.87	0.84|0.90	3|0.8685	0.898		0.90
amyloid	P16330	CN37_MOUSE_1	STOMP1|STOMP2	16|14	30	2	0.89	0.91|0.88	10|3	0.895	0.911	0.91
amyloid	P63011	RAB3A_MOUSE_1	STOMP2	14	14	1	0.9	0.9	5|0.8950	0.895		0.90
amyloid	P05063	ALDOC_MOUSE_1	STOMP1|STOMP2	10|12	22	2	0.88	0.85|0.89	4|0.8730	0.894		0.89
amyloid	P46460	NSF_MOUSE_1	STOMP1|STOMP2	4|24	28	2	0.46	0.03|0.89	8|0.4620	0.894		0.89
amyloid	Q9Z0E0	NCDN_MOUSE_1	STOMP1|STOMP2	8|16	24	2	0.89	0.82|0.97	2|4	0.894	0.97	0.97
amyloid	Q9ERD7	TBB3_MOUSE_1	STOMP1|STOMP2	9|29	38	2	0.89	0.77|1.00	1|4	0.886	1	1.00
amyloid	Q02053	UBA1_MOUSE_1	STOMP1|STOMP2	9|13	22	2	0.89	0.81|0.95	3|4	0.8775	0.947	0.95
amyloid	P08551	NFL_MOUSE_1	STOMP1|STOMP2	3|16	19	2	0.45	0.06|0.87	5|0.4675	0.873		0.87
amyloid	P63044	VAMP2_MOUSE_1	STOMP1|STOMP2	3|10	13	2	0.68	0.51|0.87	2|0.6890	0.87		0.87
amyloid	P62991	RL40_MOUSE_1	STOMP2	9	9	1	0.87	0.87	3|0.8660	0.866		0.87
amyloid	Q8BYI9	TENR_MOUSE_1	STOMP1|STOMP2	9|21	30	2	0.6	0.32|0.86	8|0.5890	0.862		0.86
amyloid	P18872	GNAO_MOUSE_1	STOMP1|STOMP2	10|23	33	2	0.86	0.72|0.98	4|5	0.8515	0.981	0.98
amyloid	Q8C8R3	ANK2_MOUSE_1	STOMP1|STOMP2	12|9	21	2	0.76	0.85|0.70	2|0.7735	0.85		0.85
amyloid	Q8BFZ3	ACTBL_MOUSE_1	STOMP1|STOMP2	17|26	43	2	0.84	0.73|0.96	5|15	0.8455	0.96	0.96
amyloid	Q7TSJ2	MAP6_MOUSE_1	STOMP1|STOMP2	4|21	25	2	0.47	0.04|0.84	7|0.4430	0.844		0.84
amyloid	Q7TQI3	OTUB1_MOUSE_1	STOMP1|STOMP2	2|9	11	2	0.58	0.36|0.84	1|0.6000	0.842		0.84
amyloid	Q9Z2Q6	SEPT5_MOUSE_1	STOMP2	9	9	1	0.83	0.83	3|0.8260	0.826		0.83
amyloid	P61922	GABT_MOUSE_1	STOMP1|STOMP2	16|25	41	2	0.8	0.77|0.82	0.797	0.823		0.82
amyloid	Q6P8J7	KCRS_MOUSE_1	STOMP2	7	7	1	0.82	0.82	2|0.8150	0.815		0.82
amyloid	Q02248	CTNB1_MOUSE_1	STOMP1|STOMP2	12|7	19	2	0.71	0.81|0.59	4|0.7005	0.809		0.81
amyloid	P15105	GLNA_MOUSE_1	STOMP1|STOMP2	14|16	30	2	0.79	0.78|0.83	5|10	0.8065	0.832	0.83
amyloid	P17742	PPIA_MOUSE_1	STOMP2	10	10	1	0.79	0.79	0.79	0.79		0.79
amyloid	Q76MZ3	2AAA_MOUSE_1	STOMP1|STOMP2	4|11	15	2	0.6	0.39|0.79	3|0.5900	0.789		0.79

Bait	Prey	PreyGene	IP	Spec	Spec Sum	Num Rep	Prob	iProb	Ctrl Counts	AvgP	MaxP	SAINT score
amyloid	P62880	GBB2_MOUSE_1	STOMP1|STOMP2	10|17	27	2	0.75	0.73|0.79	0.7575	0.785		0.79
amyloid	P62814	VATB2_MOUSE_1	STOMP1|STOMP2	8|55	63	2	0.47	0.21|0.78	0.496	0.78		0.78
amyloid	P50518	VATE1_MOUSE_1	STOMP2	10	10	1	0.77	0.77	0.766	0.766		0.77
amyloid	P62874	GBB1_MOUSE_1	STOMP1|STOMP2	6|22	28	2	0.59	0.42|0.76	0.5885	0.761		0.76
amyloid	P31324	KAP3_MOUSE_1	STOMP2	16	16	1	0.75	0.75	0.752	0.752		0.75
amyloid	P61982	1433G_MOUSE_1	STOMP2	11	11	1	0.75	0.75	0.748	0.748		0.75
amyloid	P35486	ODPA_MOUSE_1	STOMP2	12	12	1	0.74	0.74	0.737	0.737		0.74
amyloid	P99029	PRDX5_MOUSE_1	STOMP2	8	8	1	0.74	0.74	0.737	0.737		0.74
amyloid	Q9DB77	QCR2_MOUSE_1	STOMP2	14	14	1	0.74	0.74	0.736	0.736		0.74
amyloid	Q9CQV8	1433B_MOUSE_1	STOMP2	10	10	1	0.74	0.74	0.735	0.735		0.74
amyloid	Q61553	FSCN1_MOUSE_1	STOMP1|STOMP2	2|6	8	2	0.47	0.24|0.72	2|0.4795	0.722		0.72
amyloid	P02088	HBB1_MOUSE_1	STOMP1	8	8	1	0.7	0.7	4|0.7040	0.704		0.70
amyloid	P08226	APOE_MOUSE_1	STOMP1	9	9	1	0.7	0.7	0.703	0.703		0.70
amyloid	P30275	KCRU_MOUSE_1	STOMP1|STOMP2	4|14	18	2	0.42	0.14|0.70	7|0.4200	0.701		0.70
amyloid	O35129	PHB2_MOUSE_1	STOMP2	10	10	1	0.7	0.7	0.7	0.7		0.70
amyloid	Q61885	MOG_MOUSE_1	STOMP1	13	13	1	0.7	0.7	8|0.6970	0.697		0.70
amyloid	P47708	RP3A_MOUSE_1	STOMP1|STOMP2	2|6	8	2	0.52	0.28|0.70	1|0.4855	0.696		0.70
amyloid	P38647	GRP75_MOUSE_1	STOMP2	12	12	1	0.69	0.69	7|0.6900	0.69		0.69
amyloid	Q64521	GPDM_MOUSE_1	STOMP2	18	18	1	0.69	0.69	0.689	0.689		0.69
amyloid	P17426	AP2A1_MOUSE_1	STOMP2	25	25	1	0.68	0.68	0.682	0.682		0.68
amyloid	P62761	VISL1_MOUSE_1	STOMP2	7	7	1	0.68	0.68	0.682	0.682		0.68
amyloid	P48962	ADT1_MOUSE_1	STOMP1|STOMP2	26|14	40	2	0.69	0.89|0.47	14|9	0.6805	0.888	0.89
amyloid	P14094	AT1B1_MOUSE_1	STOMP1|STOMP2	5|12	17	2	0.67	0.45|0.91	2|4	0.6795	0.914	0.91
amyloid	P17156	HSP72_MOUSE_1	STOMP1|STOMP2	4|8	12	2	0.49	0.32|0.67	4|0.4975	0.674		0.67
amyloid	O88342	WDR1_MOUSE_1	STOMP1	6	6	1	0.67	0.67	2|0.6700	0.67		0.67
amyloid	P19783	COX41_MOUSE_1	STOMP2	6	6	1	0.67	0.67	0.669	0.669		0.67
amyloid	P05201	AATC_MOUSE_1	STOMP2	11	11	1	0.67	0.67	0.666	0.666		0.67
amyloid	Q9R0Y5	KAD1_MOUSE_1	STOMP2	6	6	1	0.65	0.65	0.654	0.654		0.65
amyloid	O08749	DLDH_MOUSE_1	STOMP1|STOMP2	9|14	23	2	0.62	0.58|0.65	0.6165	0.652		0.65
amyloid	P67778	PHB_MOUSE_1	STOMP2	8	8	1	0.65	0.65	0.65	0.65		0.65
amyloid	Q9EQH3	VPS35_MOUSE_1	STOMP2	6	6	1	0.65	0.65	2|0.6500	0.65		0.65
amyloid	P97807	FUMH_MOUSE_1	STOMP2	12	12	1	0.64	0.64	0.64	0.64		0.64

Bait	Prey	PreyGene	IP	Spec	Spec Sum	Num Rep	Prob	iProb	Ctrl Counts	AvgP	MaxP	SAINT score
amyloid	P17183	ENOG_MOUSE_1	STOMP1|STOMP2	3|20	23	2	0.33	0.06|0.64	11|0.3510	0.638		0.64
amyloid	Q9R0P9	UCHL1_MOUSE_1	STOMP2	7	7	1	0.63	0.63	0.632	0.632		0.63
amyloid	Q8CI94	PYGB_MOUSE_1	STOMP1|STOMP2	21|19	40	2	0.62	0.63|0.62	0.626	0.63		0.63
amyloid	Q9CPU4	MGST3_MOUSE_1	STOMP1	5	5	1	0.63	0.63	0.63	0.63		0.63
amyloid	P60335	PCBP1_MOUSE_1	STOMP2	9	9	1	0.63	0.63	0.628	0.628		0.63
amyloid	P26443	DHE3_MOUSE_1	STOMP2	12	12	1	0.63	0.63	0.627	0.627		0.63
amyloid	O88712	CTBP1_MOUSE_1	STOMP1	11	11	1	0.63	0.63	0.626	0.626		0.63
amyloid	Q60597	ODO1_MOUSE_1	STOMP1|STOMP2	10|9	19	2	0.57	0.63|0.53	7|0.5780	0.626		0.63
amyloid	P05064	ALDOA_MOUSE_1	STOMP1|STOMP2	8|29	37	2	0.59	0.25|1.00	2|6	0.6225	0.997	1.00
amyloid	Q9WV92	E41L3_MOUSE_1	STOMP1|STOMP2	7|3	10	2	0.41	0.62|0.22	3|0.4190	0.617		0.62
amyloid	P03995	GFAP_MOUSE_1	STOMP1|STOMP2	6|14	20	2	0.61	0.36|0.87	4|5	0.6155	0.867	0.87
amyloid	P16546	SPTN1_MOUSE_1	STOMP1|STOMP2	29|96	125	2	0.61	0.23|1.00	12|17	0.614	1	1.00
amyloid	Q8QZT1	THIL_MOUSE_1	STOMP1|STOMP2	2|17	19	2	0.32	0.02|0.60	10|0.3110	0.604		0.60
amyloid	Q9DB20	ATPO_MOUSE_1	STOMP2	8	8	1	0.6	0.6	5|0.6010	0.601		0.60
amyloid	P60487	PLPP_MOUSE_1	STOMP2	7	7	1	0.6	0.6	0.6	0.6		0.60
amyloid	P17751	TPIS_MOUSE_1	STOMP1|STOMP2	1|12	13	2	0.36	0.11|0.59	0.3505	0.593		0.59
amyloid	Q61206	PA1B2_MOUSE_1	STOMP2	6	6	1	0.59	0.59	0.588	0.588		0.59
amyloid	Q9D051	ODPB_MOUSE_1	STOMP1|STOMP2	2|15	17	2	0.31	0.06|0.59	9|0.3245	0.586		0.59
amyloid	Q9CQJ8	NDUB9_MOUSE_1	STOMP1|STOMP2	3|4	7	2	0.55	0.53|0.59	2|0.5570	0.585		0.59
amyloid	O54983	CRYM_MOUSE_1	STOMP2	7	7	1	0.58	0.58	0.58	0.58		0.58
amyloid	P63328	PP2BA_MOUSE_1	STOMP1|STOMP2	6|14	20	2	0.45	0.34|0.58	0.4575	0.58		0.58
amyloid	P28652	KCC2B_MOUSE_1	STOMP1|STOMP2	17|14	31	2	0.55	0.61|0.54	16|2	0.574	0.61	0.61
amyloid	O08709	PRDX6_MOUSE_1	STOMP1|STOMP2	3|6	9	2	0.48	0.41|0.57	0.4865	0.568		0.57
amyloid	P63005	LIS1_MOUSE_1	STOMP1	4	4	1	0.56	0.56	2|0.5610	0.561		0.56
amyloid	P63168	DYL1_MOUSE_1	STOMP2	3	3	1	0.56	0.56	0.558	0.558		0.56
amyloid	Q9DBJ1	PGAM1_MOUSE_1	STOMP1|STOMP2	3|8	11	2	0.45	0.33|0.56	0.445	0.558		0.56
amyloid	P20357	MTAP2_MOUSE_1	STOMP1|STOMP2	7|9	16	2	0.48	0.43|0.56	4|0.4910	0.556		0.56
amyloid	O88935	SYN1_MOUSE_1	STOMP1|STOMP2	8|26	34	2	0.56	0.15|0.96	2|11	0.555	0.964	0.96
amyloid	Q60829	PPR1B_MOUSE_1	STOMP2	5	5	1	0.56	0.56	0.555	0.555		0.56
amyloid	P97427	DPYL1_MOUSE_1	STOMP1|STOMP2	2|8	10	2	0.36	0.15|0.55	5|0.3525	0.553		0.55
amyloid	Q9CQ69	QCR8_MOUSE_1	STOMP1|STOMP2	1|3	4	2	0.46	0.38|0.55	0.4635	0.55		0.55
amyloid	O70589	CSKP_MOUSE_1	STOMP1|STOMP2	2|5	7	2	0.42	0.31|0.55	1|0.4265	0.547		0.55

Bait	Prey	PreyGene	IP	Spec	Spec Sum	Num Rep	Prob	iProb	Ctrl Counts	AvgP	MaxP	SAINT score
amyloid	P47738	ALDH2_MOUSE_1	STOMP2	5	5	1	0.55	0.55	3|0.5450	0.545		0.55
amyloid	P99024	TBB5_MOUSE_1	STOMP1|STOMP2	4|20	24	2	0.31	0.08|0.54	14|0.3090	0.543		0.54
amyloid	Q7TPR4	ACTN1_MOUSE_1	STOMP1|STOMP2	4|17	21	2	0.55	0.11|0.98	2|3	0.5425	0.977	0.98
amyloid	P62137	PP1A_MOUSE_1	STOMP2	7	7	1	0.54	0.54	0.538	0.538		0.54
amyloid	Q810U3	NFASC_MOUSE_1	STOMP1|STOMP2	3|8	11	2	0.36	0.16|0.54	4|0.3470	0.538		0.54
amyloid	Q64475	H2B1B_MOUSE_1	STOMP1|STOMP2	5|2	7	2	0.44	0.54|0.30	3|0.4205	0.537		0.54
amyloid	Q9D6F9	TBB4A_MOUSE_1	STOMP1|STOMP2	4|13	17	2	0.51	0.19|0.87	2|6	0.533	0.873	0.87
amyloid	Q9D6R2	IDH3A_MOUSE_1	STOMP1|STOMP2	2|10	12	2	0.53	0.15|0.91	2|2	0.533	0.912	0.91
amyloid	Q9QYR6	MAP1A_MOUSE_1	STOMP1|STOMP2	11|17	28	2	0.37	0.22|0.53	11|0.3780	0.533		0.53
amyloid	P14152	MDHC_MOUSE_1	STOMP2	7	7	1	0.53	0.53	0.532	0.532		0.53
amyloid	P60710	ACTB_MOUSE_1	STOMP1|STOMP2	14|40	54	2	0.54	0.11|0.95	5|22	0.5305	0.949	0.95
amyloid	P63330	PP2AA_MOUSE_1	STOMP2	7	7	1	0.53	0.53	0.526	0.526		0.53
amyloid	P62835	RAP1A_MOUSE_1	STOMP2	4	4	1	0.53	0.53	0.525	0.525		0.53
amyloid	Q8VDQ8	SIR2_MOUSE_1	STOMP1	4	4	1	0.51	0.51	2|0.5110	0.511		0.51
amyloid	O08553	DPYL2_MOUSE_1	STOMP1|STOMP2	20|76	96	2	0.51	0.03|0.99	8|23	0.5105	0.993	0.99
amyloid	Q61765	K1H1_MOUSE_1	STOMP2	8	8	1	0.51	0.51	0.506	0.506		0.51
amyloid	P01942	HBA_MOUSE_1	STOMP1	3	3	1	0.5	0.5	0.503	0.503		0.50
amyloid	P16858	G3P_MOUSE_1	STOMP1|STOMP2	5|28	33	2	0.29	0.09|0.50	20|0.2955	0.501		0.50
amyloid	Q68FD5	CLH1_MOUSE_1	STOMP1|STOMP2	21|114	135	2	0.45	0.00|1.00	6|40	0.5	1	1.00
amyloid	P17182	ENOA_MOUSE_1	STOMP1|STOMP2	5|46	51	2	0.52	0.00|1.00	3|13	0.4995	0.996	1.00
amyloid	P60904	DNJC5_MOUSE_1	STOMP1	4	4	1	0.5	0.5	0.498	0.498		0.50
amyloid	P12023	A4_MOUSE_6	STOMP1|STOMP2	30|4	34	2	0.53	0.95|0.05	4|5	0.4965	0.948	0.95
amyloid	P61264	STX1B_MOUSE_1	STOMP2	4	4	1	0.5	0.5	3|0.4960	0.496		0.50
amyloid	P68254	1433T_MOUSE_1	STOMP2	5	5	1	0.5	0.5	0.496	0.496		0.50
amyloid	Q2M3X8	PHAR1_MOUSE_1	STOMP1	10	10	1	0.5	0.5	0.496	0.496		0.50
amyloid	Q7TMM9	TBB2A_MOUSE_1	STOMP1|STOMP2	65|179	244	2	0.51	0.05|0.94	23|116	0.4935	0.942	0.94
amyloid	P05202	AATM_MOUSE_1	STOMP1|STOMP2	8|28	36	2	0.51	0.08|0.90	7|12	0.491	0.9	0.90
amyloid	P62962	PROF1_MOUSE_1	STOMP2	3	3	1	0.49	0.49	0.491	0.491		0.49
amyloid	Q91V41	RAB14_MOUSE_1	STOMP2	4	4	1	0.49	0.49	0.49	0.49		0.49
amyloid	P68372	TBB4B_MOUSE_1	STOMP1|STOMP2	16|48	64	2	0.48	0.04|0.92	7|32	0.4825	0.921	0.92
amyloid	P84244	H33_MOUSE_1	STOMP1|STOMP2	1|3	4	2	0.36	0.26|0.48	0.3655	0.476		0.48
amyloid	Q8BGD5	CPT1C_MOUSE_1	STOMP1	4	4	1	0.47	0.47	1|0.4740	0.474		0.47

Bait	Prey	PreyGene	IP	Spec	Spec Sum	Num Rep	Prob	iProb	Ctrl Counts	AvgP	MaxP	SAINT score
amyloid	P56399	UBP5_MOUSE_1	STOMP2	14	14	1	0.47	0.47	0.472	0.472		0.47
amyloid	Q91YT0	NDUV1_MOUSE_1	STOMP2	7	7	1	0.47	0.47	0.472	0.472		0.47
amyloid	P42669	PURA_MOUSE_1	STOMP2	10	10	1	0.47	0.47	8|0.4700	0.47		0.47
amyloid	P20029	GRP78_MOUSE_1	STOMP2	11	11	1	0.47	0.47	9|0.4690	0.469		0.47
amyloid	P70296	PEBP1_MOUSE_1	STOMP2	4	4	1	0.46	0.46	3|0.4640	0.464		0.46
amyloid	Q03265	ATPA_MOUSE_1	STOMP1|STOMP2	13|57	70	2	0.48	0.03|0.90	9|36	0.464	0.903	0.90
amyloid	Q61696	HS71A_MOUSE_1	STOMP1|STOMP2	2|8	10	2	0.45	0.14|0.78	2|4	0.4585	0.781	0.78
amyloid	P14231	AT1B2_MOUSE_1	STOMP2	5	5	1	0.46	0.46	0.457	0.457		0.46
amyloid	P62259	1433E_MOUSE_1	STOMP2	5	5	1	0.46	0.46	0.456	0.456		0.46
amyloid	Q9DBG3	AP2B1_MOUSE_1	STOMP1|STOMP2	8|17	25	2	0.34	0.20|0.46	0.328	0.456		0.46
amyloid	Q8BMF4	ODP2_MOUSE_1	STOMP1|STOMP2	4|8	12	2	0.36	0.24|0.45	6|0.3480	0.453		0.45
amyloid	P61979	HNRPK_MOUSE_1	STOMP1|STOMP2	2|8	10	2	0.45	0.16|0.74	5|1	0.449	0.741	0.74
amyloid	Q6X893	CTL1_MOUSE_1	STOMP1	4	4	1	0.44	0.44	2|0.4440	0.444		0.44
amyloid	Q91V61	SFXN3_MOUSE_1	STOMP2	4	4	1	0.44	0.44	3|0.4410	0.441		0.44
amyloid	P12960	CNTN1_MOUSE_1	STOMP1|STOMP2	15|14	29	2	0.43	0.44|0.43	0.4335	0.439		0.44
amyloid	Q99JR1	SFXN1_MOUSE_1	STOMP2	5	5	1	0.44	0.44	0.436	0.436		0.44
amyloid	P56480	ATPB_MOUSE_1	STOMP1|STOMP2	22|80	102	2	0.41	0.03|0.83	7|56	0.431	0.83	0.83
amyloid	P60202	MYPR_MOUSE_1	STOMP1|STOMP2	82|10	92	2	0.45	0.85|0.01	36|10	0.4305	0.85	0.85
amyloid	O55131	SEPT7_MOUSE_1	STOMP2	7	7	1	0.42	0.42	0.424	0.424		0.42
amyloid	Q91WD5	NDUS2_MOUSE_1	STOMP1	3	3	1	0.42	0.42	2|0.4240	0.424		0.42
amyloid	Q9DB05	SNAA_MOUSE_1	STOMP1|STOMP2	2|5	7	2	0.31	0.18|0.42	0.3035	0.423		0.42
amyloid	O08756	HCD2_MOUSE_1	STOMP2	4	4	1	0.41	0.41	0.412	0.412		0.41
amyloid	Q61481	PDE1A_MOUSE_1	STOMP1	8	8	1	0.41	0.41	0.41	0.41		0.41
amyloid	P21279	GNAQ_MOUSE_1	STOMP1|STOMP2	2|6	8	2	0.27	0.15|0.41	0.2805	0.408		0.41
amyloid	P26883	FKB1A_MOUSE_1	STOMP2	2	2	1	0.41	0.41	0.407	0.407		0.41
amyloid	Q8R5J9	PRAF3_MOUSE_1	STOMP1	3	3	1	0.41	0.41	0.407	0.407		0.41
amyloid	Q9QXV0	PCSK1_MOUSE_1	STOMP2	3	3	1	0.41	0.41	0.407	0.407		0.41
amyloid	P10852	4F2_MOUSE_1	STOMP2	7	7	1	0.41	0.41	0.405	0.405		0.41
amyloid	P80315	TCPD_MOUSE_1	STOMP2	7	7	1	0.41	0.41	0.405	0.405		0.41
amyloid	Q9WUA3	K6PP_MOUSE_1	STOMP2	10	10	1	0.4	0.4	0.401	0.401		0.40
amyloid	P08249	MDHM_MOUSE_1	STOMP1|STOMP2	7|34	41	2	0.39	0.06|0.73	2|30	0.3965	0.733	0.73
amyloid	P56812	PDCD5_MOUSE_1	STOMP2	2	2	1	0.39	0.39	0.39	0.39		0.39

Bait	Prey	PreyGene	IP	Spec	Spec Sum	Num Rep	Prob	iProb	Ctrl Counts	AvgP	MaxP	SAINT score
amyloid	Q64467	G3PT_MOUSE_1	STOMP1|STOMP2	5|6	11	2	0.39	0.35|0.42	2|5	0.389	0.424	0.42
amyloid	Q9JM14	NT5C_MOUSE_1	STOMP2	3	3	1	0.39	0.39	0.389	0.389		0.39
amyloid	Q61316	HSP74_MOUSE_1	STOMP2	4	4	1	0.39	0.39	2|0.3880	0.388		0.39
amyloid	P11983	TCPA_MOUSE_1	STOMP2	7	7	1	0.38	0.38	0.384	0.384		0.38
amyloid	Q99NE5	RIMS1_MOUSE_1	STOMP1|STOMP2	4|1	5	2	0.27	0.38|0.16	2|0.2725	0.383		0.38
amyloid	P47857	K6PF_MOUSE_1	STOMP1|STOMP2	3|12	15	2	0.24	0.07|0.38	0.224	0.376		0.38
amyloid	Q8K310	MATR3_MOUSE_1	STOMP1|STOMP2	14|19	33	2	0.29	0.22|0.38	17|0.2960	0.375		0.38
amyloid	Q9WTJ4	FIZ1_MOUSE_1	STOMP1	3	3	1	0.37	0.37	2|0.3730	0.373		0.37
amyloid	Q61598	GDIB_MOUSE_1	STOMP2	6	6	1	0.37	0.37	0.368	0.368		0.37
amyloid	Q99KI0	ACON_MOUSE_1	STOMP1|STOMP2	23|30	53	2	0.36	0.30|0.42	9|29	0.361	0.421	0.42
amyloid	P48318	DCE1_MOUSE_1	STOMP1|STOMP2	2|8	10	2	0.22	0.08|0.36	0.22	0.36		0.36
amyloid	P10126	EF1A1_MOUSE_1	STOMP1|STOMP2	4|6	10	2	0.31	0.25|0.36	0.306	0.358		0.36
amyloid	Q8CAQ8	IMMT_MOUSE_1	STOMP2	9	9	1	0.36	0.36	0.357	0.357		0.36
amyloid	Q8BLK3	LSAMP_MOUSE_1	STOMP1	4	4	1	0.36	0.36	0.356	0.356		0.36
amyloid	Q8R5H6	WASF1_MOUSE_1	STOMP1|STOMP2	7|7	14	2	0.35	0.36|0.34	0.3485	0.356		0.36
amyloid	Q9QYG0	NDRG2_MOUSE_1	STOMP1|STOMP2	3|5	8	2	0.29	0.24|0.36	0.2965	0.356		0.36
amyloid	O35643	AP1B1_MOUSE_1	STOMP1|STOMP2	3|13	16	2	0.2	0.06|0.35	0.2045	0.352		0.35
amyloid	P62631	EF1A2_MOUSE_1	STOMP1|STOMP2	2|6	8	2	0.25	0.12|0.35	0.2335	0.352		0.35
amyloid	P63085	MK01_MOUSE_1	STOMP2	5	5	1	0.35	0.35	0.351	0.351		0.35
amyloid	Q8K1M6	DNM1L_MOUSE_1	STOMP1|STOMP2	3|10	13	2	0.22	0.08|0.35	0.2165	0.35		0.35
amyloid	Q9Z2I9	SUCB1_MOUSE_1	STOMP2	5	5	1	0.34	0.34	0.344	0.344		0.34
amyloid	O35098	DPYL4_MOUSE_1	STOMP2	7	7	1	0.34	0.34	0.337	0.337		0.34
amyloid	Q62283	TSN7_MOUSE_1	STOMP1|STOMP2	3|3	6	2	0.34	0.32|0.34	0.3295	0.336		0.34
amyloid	P18760	COF1_MOUSE_1	STOMP1|STOMP2	2|2	4	2	0.3	0.34|0.29	2|0.3115	0.335		0.34
amyloid	Q9CR62	M2OM_MOUSE_1	STOMP1|STOMP2	2|4	6	2	0.25	0.18|0.33	0.257	0.334		0.33
amyloid	Q01853	TERA_MOUSE_1	STOMP2	9	9	1	0.32	0.32	0.32	0.32		0.32
amyloid	P27773	PDIA3_MOUSE_1	STOMP2	6	6	1	0.32	0.32	0.317	0.317		0.32
amyloid	P12382	K6PL_MOUSE_1	STOMP2	8	8	1	0.32	0.32	0.316	0.316		0.32
amyloid	Q9R0P3	ESTD_MOUSE_1	STOMP2	3	3	1	0.32	0.32	0.315	0.315		0.32
amyloid	Q9JI91	ACTN2_MOUSE_1	STOMP2	4	4	1	0.31	0.31	3|0.3070	0.307		0.31
amyloid	O70305	ATX2_MOUSE_1	STOMP1	3	3	1	0.31	0.31	2|0.3050	0.305		0.31
amyloid	P06745	G6PI_MOUSE_1	STOMP2	6	6	1	0.3	0.3	0.299	0.299		0.30

Bait	Prey	PreyGene	IP	Spec	Spec Sum	Num Rep	Prob	iProb	Ctrl Counts	AvgP	MaxP	SAINT score
amyloid	P48320	DCE2_MOUSE_1	STOMP2	6	6	1	0.3	0.3	0.295	0.295		0.30
amyloid	P08752	GNAI2_MOUSE_1	STOMP1|STOMP2	3|4	7	2	0.27	0.22|0.29	0.2565	0.292		0.29
amyloid	Q3UM45	PP1R7_MOUSE_1	STOMP2	4	4	1	0.29	0.29	0.287	0.287		0.29
amyloid	P14206	RSSA_MOUSE_1	STOMP2	3	3	1	0.28	0.28	0.28	0.28		0.28
amyloid	Q9JIS5	SV2A_MOUSE_1	STOMP1	7	7	1	0.27	0.27	0.271	0.271		0.27
amyloid	P19157	GSTP1_MOUSE_1	STOMP2	2	2	1	0.27	0.27	2|0.2680	0.268		0.27
amyloid	P40124	CAP1_MOUSE_1	STOMP1|STOMP2	1|5	6	2	0.17	0.07|0.26	0.1655	0.262		0.26
amyloid	Q8BGZ1	HPCL4_MOUSE_1	STOMP2	2	2	1	0.26	0.26	0.261	0.261		0.26
amyloid	Q9DD18	DTD1_MOUSE_1	STOMP2	2	2	1	0.26	0.26	2|0.2610	0.261		0.26
amyloid	Q8BWF0	SSDH_MOUSE_1	STOMP2	5	5	1	0.26	0.26	0.26	0.26		0.26
amyloid	Q9Z1N5	DX39B_MOUSE_1	STOMP2	4	4	1	0.26	0.26	0.256	0.256		0.26
amyloid	O35526	STX1A_MOUSE_1	STOMP1|STOMP2	2|3	5	2	0.22	0.17|0.26	0.214	0.255		0.26
amyloid	P68368	TBA4A_MOUSE_1	STOMP1|STOMP2	8|20	28	2	0.25	0.10|0.40	4|16	0.251	0.404	0.40
amyloid	Q9WVT6	CAH14_MOUSE_1	STOMP1	3	3	1	0.25	0.25	0.246	0.246		0.25
amyloid	Q922J6	TSN2_MOUSE_1	STOMP1	2	2	1	0.24	0.24	0.243	0.243		0.24
amyloid	P68510	1433F_MOUSE_1	STOMP2	2	2	1	0.24	0.24	0.237	0.237		0.24
amyloid	Q9WTL7	LYPA2_MOUSE_1	STOMP2	2	2	1	0.24	0.24	0.237	0.237		0.24
amyloid	P08553	NFM_MOUSE_1	STOMP1|STOMP2	7|8	15	2	0.22	0.20|0.24	0.218	0.236		0.24
amyloid	Q9WUM5	SUCA_MOUSE_1	STOMP2	2	2	1	0.23	0.23	2|0.2330	0.233		0.23
amyloid	Q641P0	ARP3B_MOUSE_1	STOMP1|STOMP2	1|4	5	2	0.16	0.08|0.23	0.153	0.226		0.23
amyloid	Q62420	SH3G2_MOUSE_1	STOMP2	3	3	1	0.22	0.22	0.224	0.224		0.22
amyloid	Q8VCD6	REEP2_MOUSE_1	STOMP2	2	2	1	0.22	0.22	0.224	0.224		0.22
amyloid	Q8BGU5	CCNY_MOUSE_1	STOMP2	3	3	1	0.22	0.22	0.218	0.218		0.22
amyloid	P20917	MAG_MOUSE_1	STOMP1|STOMP2	5|5	10	2	0.21	0.21|0.22	0.2145	0.216		0.22
amyloid	Q9QYF1	RDH11_MOUSE_1	STOMP1	2	2	1	0.22	0.22	2|0.2150	0.215		0.22
amyloid	Q8BG05	ROA3_MOUSE_1	STOMP1|STOMP2	2|2	4	2	0.22	0.21|0.21	2|1	0.2125	0.214	0.21
amyloid	Q01065	PDE1B_MOUSE_1	STOMP2	5	5	1	0.21	0.21	0.208	0.208		0.21
amyloid	Q9CWS0	DDAH1_MOUSE_1	STOMP2	2	2	1	0.2	0.2	0.204	0.204		0.20
amyloid	Q9QYX7	PCLO_MOUSE_1	STOMP1	7	7	1	0.2	0.2	4|0.2030	0.203		0.20
amyloid	Q99KJ8	DCTN2_MOUSE_1	STOMP2	3	3	1	0.2	0.2	0.201	0.201		0.20
amyloid	Q7TQD2	TPPP_MOUSE_1	STOMP1|STOMP2	3|11	14	2	0.2	0.10|0.31	2|9	0.2005	0.305	0.31
amyloid	Q6PHZ2	KCC2D_MOUSE_1	STOMP1|STOMP2	2|4	6	2	0.17	0.11|0.20	0.1545	0.2		0.20

Bait	Prey	PreyGene	IP	Spec	Spec Sum	Num Rep	Prob	iProb	Ctrl Counts	AvgP	MaxP	SAINT score
amyloid	Q8K0U4	HS12A_MOUSE_1	STOMP2	5	5	1	0.19	0.19	0.192	0.192		0.19
amyloid	Q8R081	HNRPL_MOUSE_1	STOMP1|STOMP2	7|3	10	2	0.13	0.19|0.07	7|0.1305	0.189		0.19
amyloid	Q8BVE3	VATH_MOUSE_1	STOMP2	4	4	1	0.18	0.18	0.184	0.184		0.18
amyloid	Q9JKN1	ZNT7_MOUSE_1	STOMP1	2	2	1	0.18	0.18	2|0.1830	0.183		0.18
amyloid	P04247	MYG_MOUSE_1	STOMP2	3	3	1	0.18	0.18	4|0.1820	0.182		0.18
amyloid	P19246	NFH_MOUSE_1	STOMP2	8	8	1	0.18	0.18	0.182	0.182		0.18
amyloid	Q9QZ06	TOLIP_MOUSE_1	STOMP2	2	2	1	0.18	0.18	0.181	0.181		0.18
amyloid	O35295	PURB_MOUSE_1	STOMP2	2	2	1	0.18	0.18	0.18	0.18		0.18
amyloid	Q99JY9	ARP3_MOUSE_1	STOMP2	3	3	1	0.18	0.18	0.176	0.176		0.18
amyloid	Q9Z1G3	VATC1_MOUSE_1	STOMP2	3	3	1	0.17	0.17	0.171	0.171		0.17
amyloid	Q9D6M3	GHC1_MOUSE_1	STOMP1	2	2	1	0.17	0.17	0.17	0.17		0.17
amyloid	P48453	PP2BB_MOUSE_1	STOMP2	4	4	1	0.16	0.16	0.164	0.164		0.16
amyloid	Q8BJU0	SGTA_MOUSE_1	STOMP1	2	2	1	0.16	0.16	0.164	0.164		0.16
amyloid	O88569	ROA2_MOUSE_1	STOMP2	8	8	1	0.16	0.16	9|0.1630	0.163		0.16
amyloid	Q62188	DPYL3_MOUSE_1	STOMP2	4	4	1	0.16	0.16	0.161	0.161		0.16
amyloid	Q9WUM4	COR1C_MOUSE_1	STOMP2	3	3	1	0.16	0.16	0.161	0.161		0.16
amyloid	P35803	GPM6B_MOUSE_1	STOMP1	2	2	1	0.16	0.16	0.16	0.16		0.16
amyloid	Q9D7G0	PRPS1_MOUSE_1	STOMP2	2	2	1	0.16	0.16	0.16	0.16		0.16
amyloid	P04370	MBP_MOUSE_1	STOMP1|STOMP2	23|16	39	2	0.16	0.17|0.14	19|8	0.1575	0.172	0.17
amyloid	Q9R1T4	SEPT6_MOUSE_1	STOMP2	3	3	1	0.16	0.16	0.157	0.157		0.16
amyloid	Q91V14	S12A5_MOUSE_1	STOMP1|STOMP2	4|9	13	2	0.11	0.04|0.16	0.099	0.155		0.16
amyloid	P35762	CD81_MOUSE_1	STOMP1|STOMP2	4|2	6	2	0.13	0.15|0.09	5|0.1215	0.153		0.15
amyloid	Q922F4	TBB6_MOUSE_1	STOMP2	3	3	1	0.15	0.15	0.152	0.152		0.15
amyloid	Q61548	AP180_MOUSE_1	STOMP2	6	6	1	0.15	0.15	0.149	0.149		0.15
amyloid	Q9JII6	AK1A1_MOUSE_1	STOMP1	2	2	1	0.15	0.15	0.149	0.149		0.15
amyloid	Q8BP47	SYNC_MOUSE_1	STOMP2	4	4	1	0.15	0.15	0.147	0.147		0.15
amyloid	Q61990	PCBP2_MOUSE_1	STOMP2	2	2	1	0.15	0.15	0.146	0.146		0.15
amyloid	Q31125	S39A7_MOUSE_1	STOMP1	3	3	1	0.14	0.14	0.143	0.143		0.14
amyloid	Q3UHJ0	AAK1_MOUSE_1	STOMP2	6	6	1	0.14	0.14	0.136	0.136		0.14
amyloid	Q61644	PACN1_MOUSE_1	STOMP1|STOMP2	9|9	18	2	0.13	0.12|0.14	10|0.1295	0.136		0.14
amyloid	Q9CZ44	NSF1C_MOUSE_1	STOMP2	2	2	1	0.13	0.13	0.134	0.134		0.13
amyloid	P03911	NU4M_MOUSE_1	STOMP1	3	3	1	0.13	0.13	4|0.1330	0.133		0.13

Bait	Prey	PreyGene	IP	Spec	Spec Sum	Num Rep	Prob	iProb	Ctrl Counts	AvgP	MaxP	SAINT score
amyloid	Q640R3	HECAM_MOUSE_1	STOMP2	2	2	1	0.13	0.13	0.131	0.131		0.13
amyloid	P61161	ARP2_MOUSE_1	STOMP2	2	2	1	0.13	0.13	0.13	0.13		0.13
amyloid	Q61301	CTNA2_MOUSE_1	STOMP2	6	6	1	0.13	0.13	0.129	0.129		0.13
amyloid	Q99L43	CDS2_MOUSE_1	STOMP1	2	2	1	0.13	0.13	2|0.1290	0.129		0.13
amyloid	Q8CGK7	GNAL_MOUSE_1	STOMP2	2	2	1	0.13	0.13	0.126	0.126		0.13
amyloid	Q8BPN8	DMXL2_MOUSE_1	STOMP1|STOMP2	61|7	68	2	0.12	0.25|0.00	88|2	0.1245	0.249	0.25
amyloid	P68181	KAPCB_MOUSE_1	STOMP1	2	2	1	0.12	0.12	0.121	0.121		0.12
amyloid	P54285	CACB3_MOUSE_1	STOMP1	2	2	1	0.12	0.12	2|0.1190	0.119		0.12
amyloid	Q922H2	PDK3_MOUSE_1	STOMP2	2	2	1	0.12	0.12	0.118	0.118		0.12
amyloid	O35286	DHX15_MOUSE_1	STOMP1	15	15	1	0.12	0.12	17|0.1170	0.117		0.12
amyloid	Q8VE33	GD1L1_MOUSE_1	STOMP1|STOMP2	1|2	3	2	0.09	0.07|0.12	0.095	0.117		0.12
amyloid	Q91WC3	ACSL6_MOUSE_1	STOMP2	4	4	1	0.12	0.12	0.117	0.117		0.12
amyloid	Q9EQF6	DPYL5_MOUSE_1	STOMP2	3	3	1	0.11	0.11	0.113	0.113		0.11
amyloid	P09671	SODM_MOUSE_1	STOMP1|STOMP2	10|4	14	2	0.11	0.17|0.06	9|5	0.112	0.169	0.17
amyloid	Q921F2	TADBP_MOUSE_1	STOMP1|STOMP2	2|2	4	2	0.11	0.09|0.11	4|0.1030	0.112		0.11
amyloid	Q61699	HS105_MOUSE_1	STOMP2	5	5	1	0.11	0.11	0.11	0.11		0.11
amyloid	P54830	PTN5_MOUSE_1	STOMP2	3	3	1	0.1	0.1	0.104	0.104		0.10
amyloid	P97441	ZNT3_MOUSE_1	STOMP1	8	8	1	0.1	0.1	11|0.1000	0.1		0.10
amyloid	Q6P1F6	2ABA_MOUSE_1	STOMP2	2	2	1	0.1	0.1	0.1	0.1		0.10
amyloid	Q8CHH9	SEPT8_MOUSE_1	STOMP2	2	2	1	0.1	0.1	0.099	0.099		0.10
amyloid	Q5SRX1	TM1L2_MOUSE_1	STOMP2	2	2	1	0.1	0.1	0.098	0.098		0.10
amyloid	Q8BG39	SV2B_MOUSE_1	STOMP1	4	4	1	0.1	0.1	0.098	0.098		0.10
amyloid	Q3V0I2	PRR7_MOUSE_1	STOMP1	7	7	1	0.1	0.1	10|0.0970	0.097		0.10
amyloid	P80313	TCPH_MOUSE_1	STOMP2	2	2	1	0.09	0.09	0.094	0.094		0.09
amyloid	Q8R071	IP3KA_MOUSE_1	STOMP2	2	2	1	0.09	0.09	0.094	0.094		0.09
amyloid	P11798	KCC2A_MOUSE_1	STOMP1|STOMP2	67|42	109	2	0.1	0.10|0.09	56|21	0.093	0.101	0.10
amyloid	O54829	RGS7_MOUSE_1	STOMP2	2	2	1	0.09	0.09	0.09	0.09		0.09
amyloid	Q8CAA7	PGM2L_MOUSE_1	STOMP2	3	3	1	0.09	0.09	0.09	0.09		0.09
amyloid	Q9QUG9	GRP2_MOUSE_1	STOMP1	3	3	1	0.09	0.09	0.09	0.09		0.09
amyloid	P62806	H4_MOUSE_1	STOMP1	3	3	1	0.09	0.09	3|4	0.089	0.089	0.09
amyloid	Q9JJK2	LANC2_MOUSE_1	STOMP2	2	2	1	0.09	0.09	0.089	0.089		0.09
amyloid	O35737	HNRH1_MOUSE_1	STOMP1|STOMP2	3|4	7	2	0.08	0.06|0.09	8|0.0760	0.088		0.09

Bait	Prey	PreyGene	IP	Spec	Spec Sum	Num Rep	Prob	iProb	Ctrl Counts	AvgP	MaxP	SAINT score
amyloid	Q923T9	KCC2G_MOUSE_1	STOMP1|STOMP2	2|3	5	2	0.09	0.07|0.09	5|0.0805	0.088		0.09
amyloid	Q6P5F7	TTYH3_MOUSE_1	STOMP1|STOMP2	2|2	4	2	0.08	0.07|0.09	0.079	0.087		0.09
amyloid	Q6PH08	ERC2_MOUSE_1	STOMP1	12	12	1	0.09	0.09	25|2	0.087	0.087	0.09
amyloid	P17809	GTR1_MOUSE_1	STOMP1	2	2	1	0.09	0.09	0.086	0.086		0.09
amyloid	Q8R0S2	IQEC1_MOUSE_1	STOMP1	18	18	1	0.09	0.09	21|0.0850	0.085		0.09
amyloid	Q3UTQ8	CDKL5_MOUSE_1	STOMP1	6	6	1	0.08	0.08	8|0.0810	0.081		0.08
amyloid	Q62108	DLG4_MOUSE_1	STOMP1	4	4	1	0.08	0.08	8|0.0810	0.081		0.08
amyloid	Q9Z1W8	AT12A_MOUSE_1	STOMP2	6	6	1	0.08	0.08	0.08	0.08		0.08
amyloid	O55143	AT2A2_MOUSE_1	STOMP2	5	5	1	0.08	0.08	0.079	0.079		0.08
amyloid	P08113	ENPL_MOUSE_1	STOMP2	4	4	1	0.08	0.08	0.078	0.078		0.08
amyloid	P16388	KCNA1_MOUSE_1	STOMP1	2	2	1	0.08	0.08	0.078	0.078		0.08
amyloid	Q8K4G5	ABLM1_MOUSE_1	STOMP1	7	7	1	0.08	0.08	11|0.0780	0.078		0.08
amyloid	P24529	TY3H_MOUSE_1	STOMP2	2	2	1	0.08	0.08	0.077	0.077		0.08
amyloid	Q8C031	LRC4C_MOUSE_1	STOMP1	5	5	1	0.08	0.08	9|0.0770	0.077		0.08
amyloid	Q9D0K2	SCOT1_MOUSE_1	STOMP2	2	2	1	0.08	0.08	0.077	0.077		0.08
amyloid	Q8BUV3	GEPH_MOUSE_1	STOMP1|STOMP2	1|3	4	2	0.06	0.04|0.08	0.0565	0.076		0.08
amyloid	Q9JLM8	DCLK1_MOUSE_1	STOMP1|STOMP2	1|4	5	2	0.05	0.03|0.07	0.0525	0.073		0.07
amyloid	P68404	KPCB_MOUSE_1	STOMP1	3	3	1	0.07	0.07	0.072	0.072		0.07
amyloid	Q61151	2A5E_MOUSE_1	STOMP2	2	2	1	0.07	0.07	0.071	0.071		0.07
amyloid	Q8C3Q5	SHSA7_MOUSE_1	STOMP1	16	16	1	0.07	0.07	21|0.0710	0.071		0.07
amyloid	Q3UTJ2	SRBS2_MOUSE_1	STOMP1	13	13	1	0.07	0.07	18|0.0690	0.069		0.07
amyloid	O35927	CTND2_MOUSE_1	STOMP1	5	5	1	0.07	0.07	5|0.0670	0.067		0.07
amyloid	Q01063	PDE4D_MOUSE_1	STOMP1	3	3	1	0.07	0.07	4|0.0670	0.067		0.07
amyloid	Q505F5	LRC47_MOUSE_1	STOMP2	2	2	1	0.07	0.07	0.066	0.066		0.07
amyloid	Q5DTL9	S4A10_MOUSE_1	STOMP1	15	15	1	0.07	0.07	22|0.0660	0.066		0.07
amyloid	Q8CA95	PDE10_MOUSE_1	STOMP2	4	4	1	0.07	0.07	0.066	0.066		0.07
amyloid	Q9JJY3	NSMA2_MOUSE_1	STOMP1|STOMP2	2|2	4	2	0.06	0.07|0.06	0.062	0.066		0.07
amyloid	Q91VR5	DDX1_MOUSE_1	STOMP1|STOMP2	3|2	5	2	0.04	0.07|0.04	5|0.0510	0.065		0.07
amyloid	Q8CGY8	OGT1_MOUSE_1	STOMP1	5	5	1	0.06	0.06	0.06	0.06		0.06
amyloid	O35681	SYT3_MOUSE_1	STOMP1	3	3	1	0.06	0.06	8|0.0590	0.059		0.06
amyloid	Q9QXS6	DREB_MOUSE_1	STOMP2	2	2	1	0.06	0.06	0.059	0.059		0.06
amyloid	Q91V92	ACLY_MOUSE_1	STOMP1|STOMP2	3|4	7	2	0.04	0.04|0.06	0.0485	0.058		0.06

Bait	Prey	PreyGene	IP	Spec	Spec Sum	Num Rep	Prob	iProb	Ctrl Counts	AvgP	MaxP	SAINT score
amyloid	P0C192	LRC4B_MOUSE_1	STOMP1	2	2	1	0.06	0.06	0.055	0.055		0.06
amyloid	Q8JZQ9	EIF3B_MOUSE_1	STOMP1|STOMP2	2|2	4	2	0.05	0.06|0.04	2|0.0495	0.055		0.06
amyloid	Q8JZR6	S4A8_MOUSE_1	STOMP1	6	6	1	0.05	0.05	11|0.0540	0.054		0.05
amyloid	Q9Z2V5	HDAC6_MOUSE_1	STOMP1	7	7	1	0.05	0.05	12|0.0530	0.053		0.05
amyloid	Q8VEK3	HNRPU_MOUSE_1	STOMP1|STOMP2	4|2	6	2	0.04	0.05|0.04	9|0.0430	0.05		0.05
amyloid	Q8VIJ6	SFPQ_MOUSE_1	STOMP1|STOMP2	12|10	22	2	0.04	0.05|0.05	19|0.0465	0.048		0.05
amyloid	Q9Z268	RASL1_MOUSE_1	STOMP2	2	2	1	0.05	0.05	0.048	0.048		0.05
amyloid	Q80TZ3	AUXI_MOUSE_1	STOMP2	3	3	1	0.05	0.05	0.047	0.047		0.05
amyloid	Q811D0	DLG1_MOUSE_1	STOMP2	3	3	1	0.05	0.05	0.047	0.047		0.05
amyloid	Q8BMT4	LRC33_MOUSE_1	STOMP2	3	3	1	0.05	0.05	8|0.0470	0.047		0.05
amyloid	P10493	NID1_MOUSE_1	STOMP1	4	4	1	0.05	0.05	0.046	0.046		0.05
amyloid	Q01097	NMDE2_MOUSE_1	STOMP1	10	10	1	0.05	0.05	17|0.0450	0.045		0.05
amyloid	Q6IFZ6	K2C1B_MOUSE_1	STOMP1|STOMP2	3|6	9	2	0.05	0.04|0.05	3|14	0.0435	0.05	0.05
amyloid	Q8C419	GP158_MOUSE_1	STOMP1	3	3	1	0.04	0.04	0.043	0.043		0.04
amyloid	O54991	CNTP1_MOUSE_1	STOMP1	4	4	1	0.04	0.04	0.041	0.041		0.04
amyloid	Q80U40	RIMB2_MOUSE_1	STOMP1	3	3	1	0.04	0.04	5|0.0410	0.041		0.04
amyloid	Q810B7	SLIK5_MOUSE_1	STOMP1	3	3	1	0.04	0.04	6|0.0410	0.041		0.04
amyloid	Q8CHC4	SYNJ1_MOUSE_1	STOMP1|STOMP2	2|5	7	2	0.03	0.02|0.04	0.0305	0.041		0.04
amyloid	Q99MX7	CECR6_MOUSE_1	STOMP1	4	4	1	0.04	0.04	11|0.0410	0.041		0.04
amyloid	Q8BIZ1	ANS1B_MOUSE_1	STOMP2	3	3	1	0.04	0.04	0.04	0.04		0.04
amyloid	Q8CC35	SYNPO_MOUSE_1	STOMP2	3	3	1	0.04	0.04	0.04	0.04		0.04
amyloid	P13595	NCAM1_MOUSE_1	STOMP2	2	2	1	0.04	0.04	0.039	0.039		0.04
amyloid	P70168	IMB1_MOUSE_1	STOMP2	3	3	1	0.04	0.04	0.037	0.037		0.04
amyloid	Q3B7Z2	OSBP1_MOUSE_1	STOMP1	2	2	1	0.04	0.04	0.037	0.037		0.04
amyloid	P70175	DLG3_MOUSE_1	STOMP1	2	2	1	0.04	0.04	3|0.0360	0.036		0.04
amyloid	O35449	PRRT1_MOUSE_1	STOMP1	7	7	1	0.04	0.04	15|0.0350	0.035		0.04
amyloid	P57780	ACTN4_MOUSE_1	STOMP2	2	2	1	0.03	0.03	0.033	0.033		0.03
amyloid	P70414	NAC1_MOUSE_1	STOMP1|STOMP2	2|2	4	2	0.03	0.03|0.03	5|0.0290	0.033		0.03
amyloid	Q11011	PSA_MOUSE_1	STOMP1	2	2	1	0.03	0.03	0.032	0.032		0.03
amyloid	Q9JHU4	DYHC1_MOUSE_1	STOMP1|STOMP2	3|20	23	2	0.02	0.01|0.03	0.023	0.032		0.03
amyloid	Q9Z2D6	MECP2_MOUSE_1	STOMP1	2	2	1	0.03	0.03	8|0.0310	0.031		0.03
amyloid	O35954	PITM1_MOUSE_1	STOMP2	2	2	1	0.03	0.03	0.03	0.03		0.03

Bait	Prey	PreyGene	IP	Spec	Spec Sum	Num Rep	Prob	iProb	Ctrl Counts	AvgP	MaxP	SAINT score
amyloid	Q5DU25	IQEC2_MOUSE_1	STOMP1	31	31	1	0.03	0.03	53|0.0300	0.03		0.03
amyloid	Q9QXL2	KI21A_MOUSE_1	STOMP2	4	4	1	0.03	0.03	0.03	0.03		0.03
amyloid	P00920	CAH2_MOUSE_1	STOMP1	9	9	1	0.03	0.03	17|0.0280	0.028		0.03
amyloid	Q8CIQ7	DOCK3_MOUSE_1	STOMP1	8	8	1	0.03	0.03	13|0.0280	0.028		0.03
amyloid	Q91XM9	DLG2_MOUSE_1	STOMP1	11	11	1	0.03	0.03	23|0.0260	0.026		0.03
amyloid	P02535	K1C10_MOUSE_1	STOMP1|STOMP2	6|12	18	2	0.03	0.01|0.03	11|21	0.024	0.034	0.03
amyloid	P70704	AT8A1_MOUSE_1	STOMP1|STOMP2	3|3	6	2	0.02	0.02|0.02	0.023	0.024		0.02
amyloid	Q3UHD9	AGAP2_MOUSE_1	STOMP2	2	2	1	0.02	0.02	0.024	0.024		0.02
amyloid	P51830	ADCY9_MOUSE_1	STOMP1	2	2	1	0.02	0.02	0.023	0.023		0.02
amyloid	Q4KMM3	OXR1_MOUSE_1	STOMP2	2	2	1	0.02	0.02	0.023	0.023		0.02
amyloid	Q9WTS6	TEN3_MOUSE_1	STOMP2	6	6	1	0.02	0.02	0.022	0.022		0.02
amyloid	Q9CQZ6	NDUB3_MOUSE_1	STOMP1	6	6	1	0.02	0.02	11|0.0210	0.021		0.02
amyloid	A2AQ25	SKT_MOUSE_1	STOMP1	6	6	1	0.02	0.02	10|0.0200	0.02		0.02
amyloid	Q99K48	NONO_MOUSE_1	STOMP1|STOMP2	28|1	29	2	0.01	0.02|0.00	41|0.0120	0.02		0.02
amyloid	P14873	MAP1B_MOUSE_1	STOMP1|STOMP2	4|7	11	2	0.01	0.01|0.02	0.011	0.017		0.02
amyloid	Q8CHG7	RPGF2_MOUSE_1	STOMP1	2	2	1	0.02	0.02	0.016	0.016		0.02
amyloid	Q61292	LAMB2_MOUSE_1	STOMP1	24	24	1	0.02	0.02	48|0.0150	0.015		0.02
amyloid	Q8CGP5	H2A1F_MOUSE_1	STOMP1|STOMP2	6|6	12	2	0.01	0.02|0.01	10|6	0.0145	0.015	0.02
amyloid	P04104	K2C1_MOUSE_1	STOMP2	9	9	1	0.01	0.01	25|0.0140	0.014		0.01
amyloid	P19096	FAS_MOUSE_1	STOMP2	6	6	1	0.01	0.01	0.014	0.014		0.01
amyloid	Q08460	KCMA1_MOUSE_1	STOMP2	3	3	1	0.01	0.01	0.014	0.014		0.01
amyloid	Q6PFD5	DLGP3_MOUSE_1	STOMP1|STOMP2	21|2	23	2	0.01	0.01|0.01	42|0.0100	0.014		0.01
amyloid	P97445	CAC1A_MOUSE_1	STOMP1|STOMP2	3|1	4	2	0.01	0.01|0.01	5|0.0125	0.013		0.01
amyloid	Q02566	MYH6_MOUSE_1	STOMP2	10	10	1	0.01	0.01	51|0.0130	0.013		0.01
amyloid	Q9EQZ7	RIMS2_MOUSE_1	STOMP1	2	2	1	0.01	0.01	4|0.0120	0.012		0.01
amyloid	Q6KCD5	NIPBL_MOUSE_1	STOMP2	6	6	1	0.01	0.01	9|0.0110	0.011		0.01
amyloid	Q811P8	RHG32_MOUSE_1	STOMP1	11	11	1	0.01	0.01	26|0.0110	0.011		0.01
amyloid	Q8VED5	K2C79_MOUSE_1	STOMP1|STOMP2	2|4	6	2	0.01	0.01|0.01	4|26	0.0105	0.012	0.01
amyloid	O88737	BSN_MOUSE_1	STOMP1|STOMP2	67|12	79	2	0.01	0.01|0.01	118|0.0085	0.01		0.01
amyloid	Q6NS60	FBX41_MOUSE_1	STOMP1	11	11	1	0.01	0.01	32|0.0080	0.008		0.01
amyloid	Q0KL02	TRIO_MOUSE_1	STOMP1	4	4	1	0.01	0.01	12|0.0060	0.006		0.01
amyloid	Q3TTY5	K22E_MOUSE_1	STOMP1|STOMP2	4|8	12	2	0.01	0.00|0.01	7|24	0.0055	0.011	0.01

Bait	Prey	PreyGene	IP	Spec	Spec Sum	Num Rep	Prob	iProb	Ctrl Counts	AvgP	MaxP	SAINT score
amyloid	Q9QXS1	PLEC_MOUSE_1	STOMP2	11	11	1	0.01	0.01	0.005	0.005		0.01
amyloid	P62737	ACTA_MOUSE_1	STOMP1|STOMP2	9|38	47	2	0	0.00|0.00	65|0.0025	0.004		0.00
amyloid	Q05793	PGBM_MOUSE_1	STOMP1	6	6	1	0	0	18|0.0030	0.003		0.00
amyloid	Q61001	LAMA5_MOUSE_1	STOMP1	2	2	1	0	0	4|0.0030	0.003		0.00
amyloid	Q9JMH9	MY18A_MOUSE_1	STOMP2	2	2	1	0	0	0.003	0.003		0.00
amyloid	Q9R1P4	PSA1_MOUSE_1	STOMP1	157	157	1	0	0	165|0.0030	0.003		0.00
amyloid	A2CG49	KALRN_MOUSE_1	STOMP1	4	4	1	0	0	14|0.0020	0.002		0.00
amyloid	Q9ESE1	LRBA_MOUSE_1	STOMP2	2	2	1	0	0	0.001	0.001		0.00
amyloid	O55126	NIPS2_MOUSE_1	STOMP1	1	1	1	0	0	0	0		0.00
amyloid	O88456	CPNS1_MOUSE_1	STOMP2	1	1	1	0	0	0	0		0.00
amyloid	P20444	KPCA_MOUSE_1	STOMP2	1	1	1	0	0	0	0		0.00
amyloid	P21278	GNA11_MOUSE_1	STOMP2	1	1	1	0	0	0	0		0.00
amyloid	P46471	PRS7_MOUSE_1	STOMP2	1	1	1	0	0	0	0		0.00
amyloid	P46664	PURA2_MOUSE_1	STOMP2	1	1	1	0	0	0	0		0.00
amyloid	P48774	GSTM5_MOUSE_1	STOMP2	1	1	1	0	0	0	0		0.00
amyloid	Q3U0V1	FUBP2_MOUSE_1	STOMP2	1	1	1	0	0	0	0		0.00
amyloid	Q62418	DBNL_MOUSE_1	STOMP2	1	1	1	0	0	0	0		0.00
amyloid	Q62433	NDRG1_MOUSE_1	STOMP1	1	1	1	0	0	0	0		0.00
amyloid	Q6GSS7	H2A2A_MOUSE_1	STOMP1	1	1	1	0	0	0	0		0.00
amyloid	Q6IME9	K2C72_MOUSE_1	STOMP2	1	1	1	0	0	3|8	0	0	0.00
amyloid	Q80SU7	GVIN1_MOUSE_1	STOMP1	1	1	1	0	0	0	0		0.00
amyloid	Q8BH95	ECHM_MOUSE_1	STOMP2	1	1	1	0	0	0	0		0.00
amyloid	Q8BHZ4	ZN592_MOUSE_1	STOMP1	1	1	1	0	0	0	0		0.00
amyloid	Q8BL65	ABLM2_MOUSE_1	STOMP1	1	1	1	0	0	0	0		0.00
amyloid	Q8BVG4	DPP9_MOUSE_1	STOMP1	1	1	1	0	0	0	0		0.00
amyloid	Q8VDD5	MYH9_MOUSE_1	STOMP2	1	1	1	0	0	0	0		0.00
amyloid	Q91X97	NCALD_MOUSE_1	STOMP2	1	1	1	0	0	0	0		0.00
amyloid	Q91YE6	IPO9_MOUSE_1	STOMP2	1	1	1	0	0	0	0		0.00
amyloid	Q921M7	FA49B_MOUSE_1	STOMP1|STOMP2	1|1	2	2	0	0.00|0.00	0	0		0.00
amyloid	Q922S4	PDE2A_MOUSE_1	STOMP1	1	1	1	0	0	1|0.0000	0		0.00
amyloid	Q922U2	K2C5_MOUSE_1	STOMP1|STOMP2	6|4	10	2	0	0.00|0.00	10|69	0	0	0.00
amyloid	Q9CZ04	CSN7A_MOUSE_1	STOMP2	1	1	1	0	0	0	0		0.00

Bait	Prey	PreyGene	IP	Spec	Spec Sum	Num Rep	Prob	iProb	Ctrl Counts	AvgP	MaxP	SAINT score
amyloid	Q9CZ30	OLA1_MOUSE_1	STOMP2	1	1	1	0	0	0	0		0.00
amyloid	Q9D0L8	MCES_MOUSE_1	STOMP2	1	1	1	0	0	0	0		0.00
amyloid	Q9DCU2	PLLP_MOUSE_1	STOMP1	1	1	1	0	0	0	0		0.00
amyloid	Q9ER00	STX12_MOUSE_1	STOMP2	1	1	1	0	0	0	0		0.00
amyloid	Q9R1Q8	TAGL3_MOUSE_1	STOMP2	1	1	1	0	0	0	0		0.00
amyloid	Q9WV60	GSK3B_MOUSE_1	STOMP2	1	1	1	0	0	0	0		0.00
