# Supplementary material for: Determining composition of micron-scale protein deposits in neurodegenerative disease by spatially targeted optical microproteomics
Source: eLife. 2015 Sep 29;4:e09579. doi: 10.7554/eLife.09579 (PMC4630677; doi:10.7554/eLife.09579)
Supplement: Supplementary file 2. — Table is sorted in descending order of abundance, by normalized spectral counts. DOI: http://dx.doi.org/10.7554/eLife.09579.014 [file elife09579s002.docx]

**Supplementary File 2:**

High abundance proteins in non-plaque regions of the TgCRND8 mouse brain identified and retrieved by STOMP. Table is sorted in descending order of abundance, by normalized spectral counts.

| **Protein** | **UniProt ID** | **MW (kDa)** | **STOMP counts** | **Dark counts** |
| --- | --- | --- | --- | --- |
| Myelin basic protein | P04370 | 27.2 | 69.9 | 0.0 |
| Calcium/calmodulin-dependent protein kinase type II subunit alpha | P11798 | 54.1 | 34.2 | 3.7 |
| Myelin proteolipid protein | P60202 | 29.9 | 33.4 | 0.0 |
| Glyceraldehyde-3-phosphate dehydrogenase | P16858 | 35.7 | 30.8 | 7.0 |
| Tubulin alpha-1A chain | P16858 | 50.1 | 27.9 | 2.0 |
| Vesicle-associated membrane protein 2 | P63044 | 12.6 | 27.9 | 0.0 |
| Synapsin-1 OS=Mus musculus | O88935 | 74.1 | 26.3 | 0.0 |
| Dihydropyrimidinase-related protein 2 | O08553 | 62.3 | 24.9 | 0.0 |
| Tubulin beta-2A chain | Q7TMM9 | 49.9 | 22.0 | 6.0 |
| Tubulin beta-2C chain | P68372 | 49.8 | 19.1 | 0.0 |
| Actin, aortic smooth muscle | P62737 | 41.8 | 18.0 | 0.0 |
| Sodium/potassium-transporting ATPase subunit alpha-3 | Q6PIC6 | 111.7 | 14.8 | 0.0 |
| Glutamine synthetase | P15105 | 42.0 | 14.3 | 0.0 |
| Heterogeneous nuclear ribonucleoproteins A2/B1 | O88569 | 37.4 | 13.4 | 0.0 |
| ATP synthase subunit beta, mitochondrial | P56480 | 51.7 | 12.6 | 0.0 |
| Sodium/potassium-transporting ATPase subunit alpha-1 | Q8VDN2 | 112.5 | 12.0 | 0.0 |
| Non-POU domain-containing octamer-binding protein | Q99K48 | 54.5 | 11.0 | 0.0 |
| Calcium/calmodulin-dependent protein kinase type II subunit beta | P28652 | 60.5 | 10.8 | 0.0 |
| Guanine nucleotide-binding protein G(o) subunit alpha | P18872 | 40.0 | 10.0 | 0.0 |
| Splicing factor, proline- and glutamine-rich | Q8VIJ6 | 75.4 | 8.0 | 0.0 |
| Aconitate hydratase, mitochondrial | Q99KI0 | 82.5 | 7.9 | 0.0 |
| Syntaxin-binding protein 1 | O08599 | 67.6 | 7.4 | 0.0 |
| Neurofilament light polypeptide | P08551 | 61.4 | 7.3 | 0.0 |
| Protein shisa-7 | Q8C3Q5 | 56.4 | 7.1 | 0.0 |
| Glial fibrillary acidic protein | P03995 | 49.9 | 7.0 | 0.0 |
| Neurotrimin | Q99PJ0 | 31.7 | 6.3 | 0.0 |
| Syntaxin-1B | P61264 | 33.2 | 6.0 | 0.0 |
| ATP synthase subunit alpha, mitochondrial | Q03265 | 55.3 | 4.5 | 0.0 |
| Dynamin-1 | P39053 | 97.8 | 3.6 | 0.0 |
| Dystrobrevin alpha | Q9D2N4 | 84.1 | 2.4 | 0.0 |
| IQ motif and SEC7 domain-containing protein 2 | Q5DU25 | 161.8 | 1.5 | 0.0 |
