# Supplementary material for: Determining composition of micron-scale protein deposits in neurodegenerative disease by spatially targeted optical microproteomics
Source: eLife. 2015 Sep 29;4:e09579. doi: 10.7554/eLife.09579 (PMC4630677; doi:10.7554/eLife.09579)
Supplement: Supplementary file 3. — DOI: http://dx.doi.org/10.7554/eLife.09579.015 [file elife09579s003.docx]

**Supplementary File 3:**

**26 enriched proteins enriched in senile plaques relative to non-plaque regions detected by Liao et al. (2004) (ref. 18).**

| **Protein** | **Mouse**  **STOMP** | **Human**  **STOMP** |
| --- | --- | --- |
| collagen I, alpha-1 polypeptide | o | o |
| fibrinogen, gamma | o | o |
| ATPase, Ca++ transporting | x | o |
| heat shock 90kDa protein 1, beta | XX | x |
| coronin, actin binding protein | x | XX |
| tau | o | o |
| glial fibrillary acidic protein (GFAP) | x | XX |
| vimentin | o | o |
| 14-3-3 beta/alpha | o | o |
| 14-3-3 epsilon | o | o |
| 14-3-3 zeta | o | o |
| clathrin, heavy polypeptide 1 | XX | XX |
| dynamin 1 | XX | o |
| dynein, heavy polypeptide 1 | x | x |
| phosphofructokinase | x | o |
| amyloid beta-peptide | XX | XX |
| antitrypsin | o | o |
| ATPase, H+ transporting, lysosomal V0 subunit A | XX | XX |
| ATPase, H+ transporting, lysosomal V1 subunit B | XX | XX |
| ATPase, H+ transporting, lysosomal V1 subunit D | o | x |
| ATPase, H+ transporting, lysosomal V1 subunit E | x | o |
| cathepsin D | o | o |
| cystatin B | o | o |
| cystatin C | o | o |
| ubiquitin-activating enzyme E1 | XX | XX |
| vacuolar ATPase subunit H | o | o |
| **Present and enriched over dark control** | **7** | **7** |
| **Present, not sufficiently enriched over dark control** | **6** | **3** |
| Not detected in STOMP samples | 13 | 16 |

XX – present in STOMP hits, enriched over dark control

x – detected in STOMP sample(s), but not enriched

o – not detected by STOMP
