# Supplementary material for: Determining composition of micron-scale protein deposits in neurodegenerative disease by spatially targeted optical microproteomics
Source: eLife. 2015 Sep 29;4:e09579. doi: 10.7554/eLife.09579 (PMC4630677; doi:10.7554/eLife.09579)
Supplement: Supplementary file 4. — DOI: http://dx.doi.org/10.7554/eLife.09579.016 [file elife09579s004.docx]

**Supplementary File 4:**

**Antibodies used for immunofluorescence and immunohistochemistry.**

| **Antibody** | **Type** | **Source** | **Dilution (frozen)** | **Dilution (formalin)** |
| --- | --- | --- | --- | --- |
| ApoE | Mo mAb | Abcam ab1906 | 1:125 | 1:125 |
| ApoJ | Rb pAb | Abcam ab42673 | 1:80 | 1:80 |
| ATP6V1B2 | Rb pAb | Abcam ab73404 | 1:300 |  |
| β-amyloid peptide (6E10) | Mo mAb | Sigma A1474 | 1:300 |  |
| GFAP | Rb pAb | Abcam ab7260 | 1:400 |  |
| SNAP25 (SMI 81) | Mo mAb | Abcam ab24737 | 1:200 | 1:2000 |
| synapsin 1 | Rb pAb | Chemicon (Millipore) MAB355 | 1:300 |  |
| synaptophysin (SY 38) | Mo mAb | Abcam ab8049 | 1:20 |  |
| synaptophysin | Rb pAb | EMD Millipore ab9272 |  | 1:500 |
| syntaxin 1 | Mo mAb | Sigma S0664 | 1:1000 |  |
| α-synuclein | Rb mAb | Abcam ab138501 | 1:300 |  |
| Tau | Mo mAb | In-house |  | 1:100 |
| VAMP2 | Rb pAb | Abcam 70222 | 1:300 | 1:3000 |
| Hexihistidine | Mo mAb | GenScript A00186 | 1:100 |  |
| anti-mouse Texas Red | Go pAb | Molecular Probes (Life Tech.) T-862 | 1:200 |  |
| anti-mouse Alexa 488 | Go pAb | Molecular Probes (Life Tech.) A-11001 | 1:200 |  |
| anti-rabbit Texas Red | Go pAb | Molecular Probes (Life Tech.) T-2767 | 1:200 |  |

Mo = mouse, Rb = rabbit, Go = goat, mAb = monoclonal antibody, pAb = polyclonal antibody.

**The STOMP macro:**

The STOMP macro was developed based on code from a custom photobleaching tool (Pham HH, Gourevich, I, Jonkman JEN, Kumacheva E (2007) “Polymer nanostructured material for the recording of biometric features” *Journal of Materials Chemistry* **17**:523-6.) The current version has been tested on LSM 700- and 800-series upright and inverted confocal instruments. The STOMP macro is a Visual Basic for Applications (VBA) macro that can be called from the standard Carl Zeiss ZEN (Black edition) microscope control software. The ZEN Visual Basic Macro package is *not* required to run the macro, but it is necessary if individuals users wish to edit or customize the STOMP tool.

# Generating the STOMP mask file

We use ImageJ to create the image masks used for STOMP, as recent versions of ImageJ include both the native ability to read Zeiss’ .lsm image file format (earlier versions of ImageJ can use a plugin) and the ability to write ‘text image’ formatted files.

# STOMP macro interface

**Mask file name**. Enter the full path and file name (including the file extension—typically .txt) for the mask file.

**% laser power**. This field sets the transmission of the AOTF/AOM (acousto-optic tunable filter or acousto-optic modulator), controlling the intensity of laser light irradiating the specimen. This is expressed as a percentage; it does not compensate for changes in laser intensity (which may be significant when changing the operating wavelength of a tunable femtosecond laser). Always determine safe power levels on a test specimen.

**Iterations**. This field controls the number of passes the macro will make over each pixel in the target mask. We have found that local, rapid sample heating (and subsequent sample damage and distortion) can occur if the laser remains on one spot for an extended period of time (more than a few milliseconds). Repeated short laser exposures allow the safe use of higher powers than are possible with a single long exposure. As well, there may be an advantage to allowing fresh unbound photo-tag to diffuse into the volume of interest between passes with the laser.

**Laser line**. This drop-down box selects the laser wavelength used for STOMP. This list is created when the macro is started; it will not reflect changes in the available wavelengths if different lasers are turned on or off, or if a laser is retuned to a new wavelength.

**Mirror delay**. This is the time interval allowed, in milliseconds (ms), for the scanning mirrors to come to rest after a large adjustment in their position (e.g. when moving between one region of interest and another).

**AOTF delay**. This is the time interval allowed, in milliseconds (ms), for the AOTF to shutter the laser on or off before re-aiming the scanning mirrors. To move from one region of interest to another, the following events and delays take place:

- Scan to last pixel of ROI 1.

- Command AOTF to close.

- Pause for AOTF delay.

- Move to first pixel of ROI 2.

- Pause for mirror delay.

- Command AOTF to open.

- Pause for AOTF delay.

- Begin scan of ROI 2.

**Pixel delay**. This is the time interval allowed, in milliseconds (ms), for the laser to dwell on each pixel in a ROI.

**Log file name**. This is a text file to which macro log entries are recorded. (The log entries for the most recent STOMP run are also displayed in the large box below.) The log file records the name of the mask file, the total number of pixels to be STOMPed (in the mask image), the laser wavelength selected (based on the setting in the “laser line” box), the AOTF transmission %, the number of iterations requested, and a date stamp for the start of the STOMP run.

At the end of each STOMP run, the log file reports the total time elapsed between the start and finish of the run, including all iterations (“Total elapsed time”) and the total time that the AOTF shutter was open (“Total beam time”)—that is, the total amount of time that photolabeling was occurring. The total amount of material labeled by STOMP should be proportional to this ‘beam’ time. (See the note below regarding complex, ‘speckled’ masks.)

**Verbose logs**. When checked, a new line will be added to the STOMP log file after iteration through the mask file, recording the iteration number and a date stamp.

If there is a significant variation in the time required to complete successive iterations through the same mask file, this may be indicative of unusual background activity on the computer (caused by other applications, antivirus scans, etc.). Shutting down other processor- or disk-intensive applications, postponing virus scans, and/or running the STOMP application at a higher priority may be required.

**Log comment**. This optional field is recorded to the log file as part of the header information for each STOMP run.

**STOMP** button. Click to start STOMPing. The macro will open and read the mask file, processing it (internally) to identify the boundaries of each region of interest within the mask and creating a list of coordinates to be targeted. If all goes well, a dialog box will appear, displaying the total number of pixels to be STOMPed in the mask file and a Yes/No option to proceed. (Depending on the size of the mask file and the speed of the computer system, it may take a few seconds to read and analyze the mask. We have noticed that the process of reading the mask file from external USB ‘thumb drives’ is sometimes quite a bit slower than reading from an internal hard drive.)

Once begun, the progress bar will show percent completion of the STOMP run (including all iterations). The status line above the progress bar shows the number of coordinates completed in the current iteration through the mask file, the total number of coordinates in the mask, the current iteration, and the total number of iterations. (The number of coordinates will typically be slightly greater than the number of pixels to be STOMPed, as extra coordinates are added to the list to mark points where the shutter is closed between ROIs.)

The clocks above the progress bar tally the total time elapsed since the beginning of the STOMP run, and the total time that the beam has been on (that is, the shutter open) during the run. If the beam-on time is *not* a substantial fraction of the total time, then the macro is spending a lot of time shuttering the beam on and off and re-aiming the mirrors.
